# Homoharringtonine Suppresses Prostate Cancer Progression Through Modulation of the TNF/NF-κB and MAPK Signaling Pathways

**DOI:** 10.3390/cells15191788

**Published:** 2026-09-30

**Authors:** Yuzhong Chen, Sisi Huang, Dongyan Huang, Yangtao Jin, Congcong Shao, Xin Su, Rongfu Yang, Juan Jiang, Jianhui Wu

**Affiliations:** 1 NHC Key Lab of Reproduction Regulation, Shanghai Engineering Research Center of Reproductive Health Drug and Devices, Shanghai Institute for Biomedical and Pharmaceutical Technologies, Pharmacy School of Pharmaceutical Sciences Fudan University, Shanghai 200237, China; 24211150006@m.fudan.edu.cn (Y.C.); sisihuang22@m.fudan.edu.cn (S.H.); huangdongyan@sibpt.cn (D.H.); 23211150006@m.fudan.edu.cn (Y.J.); shaocongcong@sibpt.cn (C.S.); suxin@sibpt.cn (X.S.); yangrongfu@sibpt.cn (R.Y.); jiangjuan@sibpt.cn (J.J.); 2 Department of Pharmacology & Toxicology, Shanghai Institute for Biomedical and Pharmaceutical Technologies, Shanghai 200032, China

**Keywords:** natural product, homoharringtonine, prostate cancer

## Abstract

Prostate cancer (PCa) represents one of the leading causes of cancer-related mortality among men. The efficacy of its treatment is often compromised by chemotherapeutic toxicity and multidrug resistance, underscoring the urgent need to develop efficient and low-toxicity alternative therapeutic strategies. Homoharringtonine (HHT), a bioactive compound, has demonstrated clinical anticancer efficacy in hematologic malignancies. However, its role and underlying mechanisms in prostate cancer remain largely unclear. This study provides evidence that HHT suppresses prostate cancer cell proliferation and promotes cell cycle arrest by modulating the TNF/NF-κB and MAPK signaling pathways, indicating its potential as a promising natural drug candidate for PCa treatment.

## 1. Introduction

Prostate cancer (PCa) is one of the leading causes of cancer-related death in men [1]. Epidemiological surveys have shown that the global incidence of PCa is rising annually [2]. Most patients with this malignancy are diagnosed at an advanced stage and have a poor prognosis [3]. Currently, androgen deprivation therapy, postoperative radiotherapy, and chemotherapy are common treatment strategies for PCa [4,5]. However, multidrug resistance and the toxicity of chemotherapeutic agents frequently compromise treatment outcomes [6,7]. Although various chemotherapeutic drugs and targeted therapies are employed in clinical practice, the therapeutic efficacy for PCa remains unsatisfactory, particularly in patients with advanced metastatic disease [8,9,10]. Therefore, there is an urgent need for research into novel drugs for the treatment of PCa.

**Figure 1:**
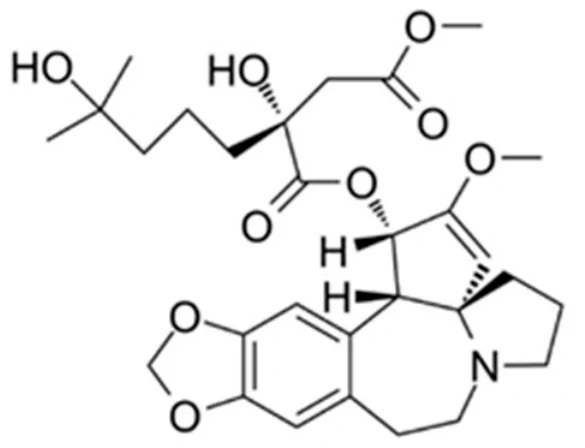
Chemical structure of Homoharringtonine (HHT).

Homoharringtonine (HHT) is an ester alkaloid extracted from plants of the Cephalotaxaceae family (Figure 1). It has been widely used in the treatment of hematologic malignancies, including chronic myeloid leukemia (CML) and acute myeloid leukemia (AML), by inhibiting the initial elongation step of protein synthesis, thereby suppressing translation [11,12,13]. Multiple studies have demonstrated that HHT also exerts anticancer effects against solid tumors. Wang et al. reported that HHT inhibits breast cancer cell growth by regulating the miR-18a-3p/AKT/mTOR signaling pathway [14]. Shi et al. demonstrated that HHT suppresses colorectal cancer growth by inhibiting EphB4 and its downstream PI3K/AKT and MAPK/ERK1/2 signaling pathways and by modulating cell cycle-related molecules and Bcl-2 mitochondrial apoptotic pathway molecules [15]. Furthermore, a recent study indicated that HHT attenuates dextran sulfate sodium (DSS)-induced colitis in mice by inhibiting the macrophage-associated NF-κB signaling pathway and M1 polarization [16]. Notably, Zou et al. recently identified an AR-positive, neuroendocrine-negative small-cell-like prostate cancer (SCLPC) subtype characterized by upregulated translation elongation and an SP1-driven signature and demonstrated that HHT, an FDA-approved translation elongation inhibitor, suppressed tumor growth in multiple CRPC models and produced clinical benefits in patients with CRPC. These findings provide direct evidence for the use of HHT in PCa, especially the CRPC/AR-low subtype [17]. These results suggest that HHT may serve as a potential candidate drug for the treatment of PCa.

This study investigated the anti-PCa activity of HHT, including its effects on cell proliferation, migration, invasion, cell cycle, and apoptosis. At the mechanistic level, we explored the roles of the TNF/NF-κB and MAPK signaling pathways in mediating HHT-induced inhibition of proliferation and induction of apoptosis. We provide evidence that HHT inhibits PCa cell growth and induces apoptosis through the regulation of signaling pathways. These findings indicate that HHT is a potential natural candidate drug for the treatment of PCa. This study supports the future application of HHT in the therapy of PCa.

## 2. Materials and Methods

### 2.1. Chemicals

HHT (CAS No: 26833-87-4, purity ≥ 98%) was purchased from Hepai Biotechnology Co., Ltd. (Shanghai, China). The Cell Counting Kit-8 (CCK-8) and cell cycle detection kit were provided by Beyotime Biotechnology Co., Ltd. (Shanghai, China). The Annexin V-FITC/PI Apoptosis Detection Kit was obtained from Yeason Biotechnology (Shanghai, China).

### 2.2. Cell Culture

PC-3 and LNCaP cells were obtained from the Cell Bank of the Chinese Academy of Sciences. Human normal prostatic stromal immortalized cells (WPMY-1) and human prostate fibroblasts (HPRF) were purchased from Shanghai Zhongqiao Xinzhou Biotechnology Co., Ltd. (Shanghai, China) PC-3 cells were cultured in F12K medium supplemented with 10% fetal bovine serum (FBS); DU145 cells were maintained in MEM medium containing 10% FBS; LNCaP cells were maintained in RPMI-1640 medium containing 10% FBS; WPMY-1 cells were cultured in high-glucose DMEM supplemented with 10% FBS; HPRF cells were cultured in FM medium containing 2% FBS and 1% fibroblast growth supplement (FGS). All cell lines were incubated at 37 °C in a humidified atmosphere of 5% CO_2_, and all culture media were supplemented with 1% penicillin-streptomycin solution.

### 2.3. Cell Morphology

Cells were seeded in 96-well plates at densities of 8000 cells/well for PC-3 and 10,000 cells/well for LNCaP. Cells were then treated with vehicle (0.1% DMSO) or HHT (12.5–200 nM). After 48 h, morphological changes were observed and photographed under an optical microscope (Aosvl, Guangzhou, China).

### 2.4. Cell Viability Assay

Cells were seeded in 96-well plates at the following densities: PC-3 at 10,000 cells/well for 24 h, 8000 cells/well for 48 h, and 6000 cells/well for 72 h; LNCaP at 12,000 cells/well for 24 h, 10,000 cells/well for 48 h, and 8000 cells/well for 72 h; WPMY-1 at 6000 cells/well; and HPRF at 6000 cells/well. Cells were treated with vehicle (0.1% DMSO) or HHT (12.5–200 nM) for 24, 48, and 72 h. Subsequently, 10 μL of CCK-8 solution was added to each well, followed by incubation at 37 °C in the dark for 2–4 h. Absorbance was measured at 450 nm using a microplate reader (BioTek, Santa Clara, CA, USA).

### 2.5. Colony-Formation Assay

Cells were seeded in six-well plates at a density of 1 × 10^2^–5 × 10^2^ cells/mL. The cells were treated with vehicle (0.1% DMSO) or HHT (25, 50, and 100 nM) for 48 h, followed by continuous culture for 10–12 days with regular medium replacement. The cells were then fixed with 4% paraformaldehyde, stained with crystal violet, and photographed. ImageJ v1.8.0 was used for data analysis.

### 2.6. Annexin V-FITC/PI Double-Staining Apoptosis Detection

Cells were seeded in six-well plates at a density of 2.0–2.5 × 10^5^ cells/well and allowed to attach overnight. After attachment, cells were treated with vehicle (0.1% DMSO) or HHT at concentrations ranging from 12.5 to 200 nM for 48 h. Cells were then harvested, resuspended in 100 μL of 1× binding buffer (Yeasen, Shanghai, China), and stained with Annexin V-FITC and propidium iodide (PI) for 15 min at room temperature in the dark. Subsequently, samples were analyzed by flow cytometry (BD Biosciences, San Jose, CA, USA).

### 2.7. Cell Cycle Analysis

Cells were seeded in six-well plates at a density of 2.0–2.5 × 10^5^ cells/well and cultured overnight. Cells were then treated with vehicle (0.1% DMSO) or HHT at concentrations ranging from 12.5 to 200 nM for 48 h. Subsequently, cells were harvested, washed with pre-cooled PBS, and fixed with 70% ethanol overnight. On the next day, cells were resuspended in 500 μL of staining buffer containing 25 μL of propidium iodide (PI) and 10 μL of RNase A and incubated at 37 °C in the dark for 30 min. The DNA content of the cells was determined by flow cytometry, and the proportions of cells in different phases of the cell cycle were analyzed using FlowJo v10.8.1 software.

### 2.8. Wound Healing Assay

Cells were seeded in six-well plates at a density of 2.0–2.5 × 10^5^ cells/well. A sterile 200 μL pipette tip was used to create a vertical scratch across the bottom of each well. Floating cells were removed by washing with PBS, leaving a clear wound area. Cells were then treated with vehicle (0.1% DMSO) or HHT at concentrations ranging from 25 to 100 nM for 24 and 48 h. Wound closure was photographed under an optical microscope at 0, 24, and 48 h, and the scratched area was quantified using ImageJ software.

### 2.9. Transwell Assay

For migration assays, cells were seeded at a density of 5–15 × 10^4^ cells/well; for invasion assays, cells were seeded at a density of 10–30 × 10^4^ cells/well. Cells were resuspended in 100 μL of serum-free medium containing vehicle (0.1% DMSO) or final concentrations of HHT (25, 50, and 100 nM) and placed in the upper chambers of Transwell plates. For invasion assays, the upper chambers were pre-coated with Matrigel at a final concentration of 10% (this step was omitted for migration assays). The lower chambers were filled with 600 μL of culture medium supplemented with 10% FBS. After 48 h of HHT treatment, cells remaining on the upper surface of the Transwell membrane were gently removed with a cotton swab. Cells on the lower surface were fixed with 4% paraformaldehyde for 15 min at room temperature and stained with crystal violet for 20 min. Migration and invasion were then visualized and photographed under a microscope.

### 2.10. Transcriptome Sequencing

PC-3 cells were treated with vehicle (0.1% DMSO) or HHT (100 nM) for 48 h, with three biological replicates per group. Total RNA was extracted using TRIzol reagent, and RNA sequencing was performed at the Shanghai Institute of Biomedical Technology. DEGs were identified based on |log_2_ fold change| ≥ 1 and FDR ≤ 0.05, yielding 1542 DEGs in total, including 620 upregulated and 922 downregulated genes.

For enrichment analysis, a hypergeometric test was employed, with all annotated genes in the genome used as the background gene set. *p* values were adjusted by the Benjamini–Hochberg method, and q < 0.05 was considered statistically significant. All 1542 DEGs were used as the input. The analyses were conducted using the clusterProfiler package (version 4.12.0) and R software (version 4.5.0).

### 2.11. Real-Time Quantitative Polymerase Chain Reaction (RT-qPCR)

Total RNA was extracted using the RNAeasy™ Kit (R0026, Beyotime Biotechnology, Shanghai, China). RNA purity and concentration were determined using a spectrophotometer (ACT Gene, Shanghai, China). After reverse transcription, mRNA expression levels were detected using a Roche LightCycler^®^ 480 Real-Time PCR System (Roche, Rotkreuz, Switzerland). The primer sequences are listed in Table 1.

### 2.12. Statistical Analysis

All results are expressed as mean ± standard deviation (SD). Statistical analyses and graphical presentations were performed using SPSS 26.0 and GraphPad Prism 9.0 (GraphPad Software, San Diego, CA, USA). A *p* value < 0.05 was considered statistically significant.

## 3. Results

### 3.1. Effects of HHT on the Morphology of PCa Cells

**Figure 2:**
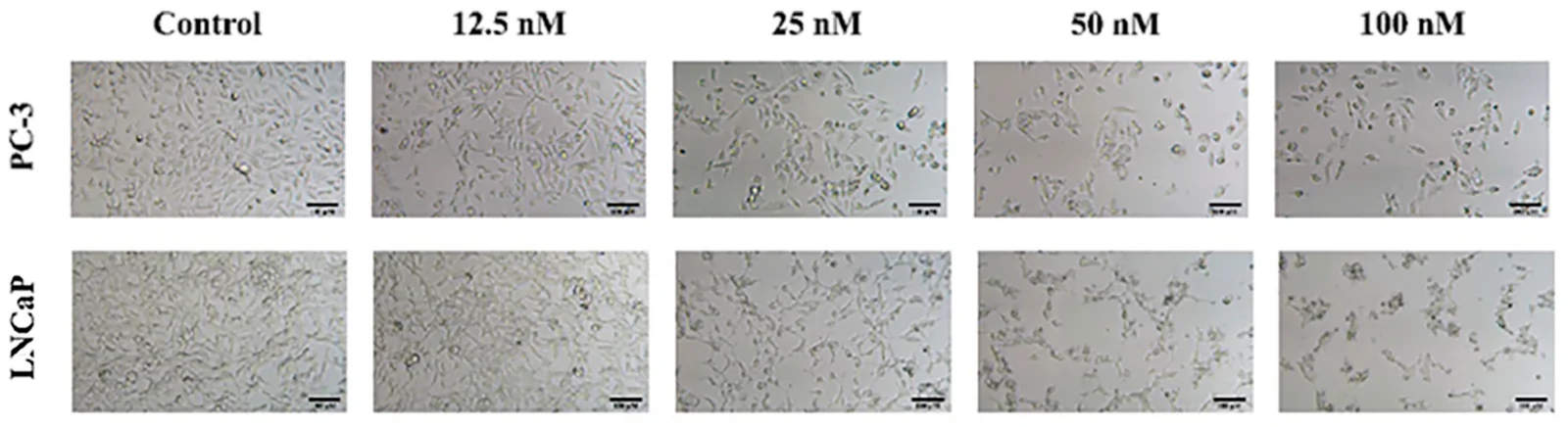
Morphological changes in PC-3 and LNCaP cells after HHT treatment (100×; scale bar, 50 μm).

After treatment with HHT (12.5–200 nM) for 48 h, concentration-dependent morphological changes were observed in both PC-3 and LNCaP cells (Figure 2). Specifically, with increasing doses, the cell contours became blurred and cell adhesion decreased.

### 3.2. Effects of HHT on PCa Cell Viability

**Figure 3:**
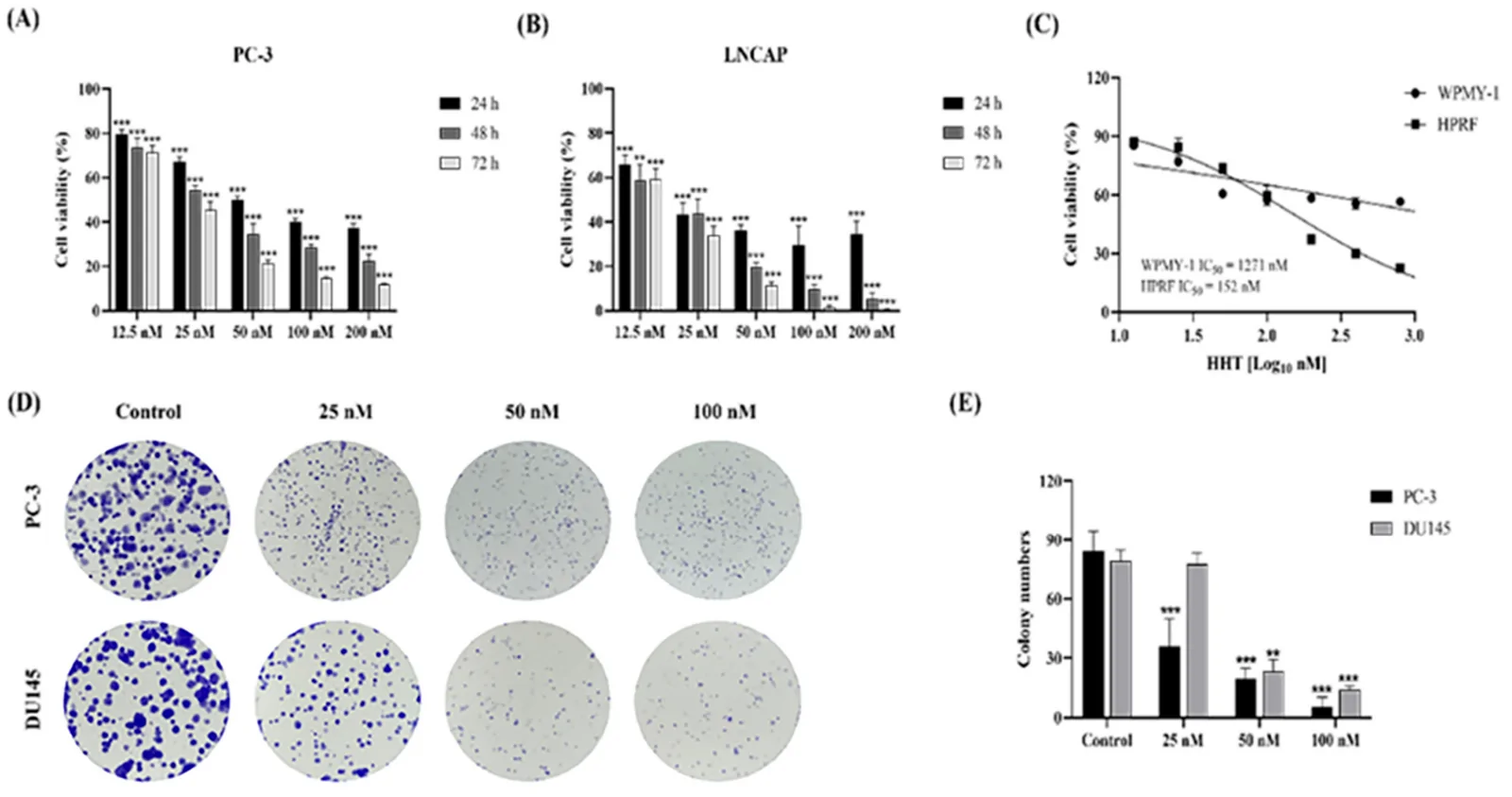
Effects of HHT on the growth of PC-3 and LNCaP PCa cells: (**A**,**B**) effect of HHT on the viability of PC-3 and LNCaP cells after 24, 48, and 72 h of treatment; (**D**) effect of HHT on the colony-forming ability of PC-3 and DU145 cells and the corresponding statistical histogram (**E**); (**C**) cell viability curves of WPMY-1 and HPRF cells after 48 h of HHT treatment. Data are presented as mean ± SD. ** *p* < 0.01,*** *p* < 0.001, *n* = 3.

The inhibitory effect of HHT on the growth of PC-3 and LNCaP cells was evaluated using the CCK-8 assay at 24, 48, and 72 h post-treatment. The colony formation assay was further employed to confirm the inhibitory effect, which showed that the number and size of PC-3 cell colonies were significantly reduced (Figure 3B). Taken together, these results demonstrate that HHT significantly inhibits the growth of PCa cells.

After 48 h of treatment, the IC_50_ values of HHT for PC-3 and LNCaP cells were 32.31 nM and 17.77 nM, respectively; after 72 h of treatment, the IC_50_ values were 22.74 nM and 16.06 nM, respectively (Table 2). HHT exhibited IC_50_ values of 152 nM and 1271 nM against HPRF and WPMY-1 cells, respectively, which were markedly higher than those against PC-3 (32.31 nM) and LNCaP (17.77 nM) at 48 h (Figure 3C). These data indicate that HHT exerts lower cytotoxicity toward normal prostate cells while selectively killing prostate cancer cells. In addition, LNCaP cells were more sensitive to HHT than PC-3 cells.

### 3.3. HHT Inhibits the Migration and Invasion of PCa Cells

**Figure 4:**
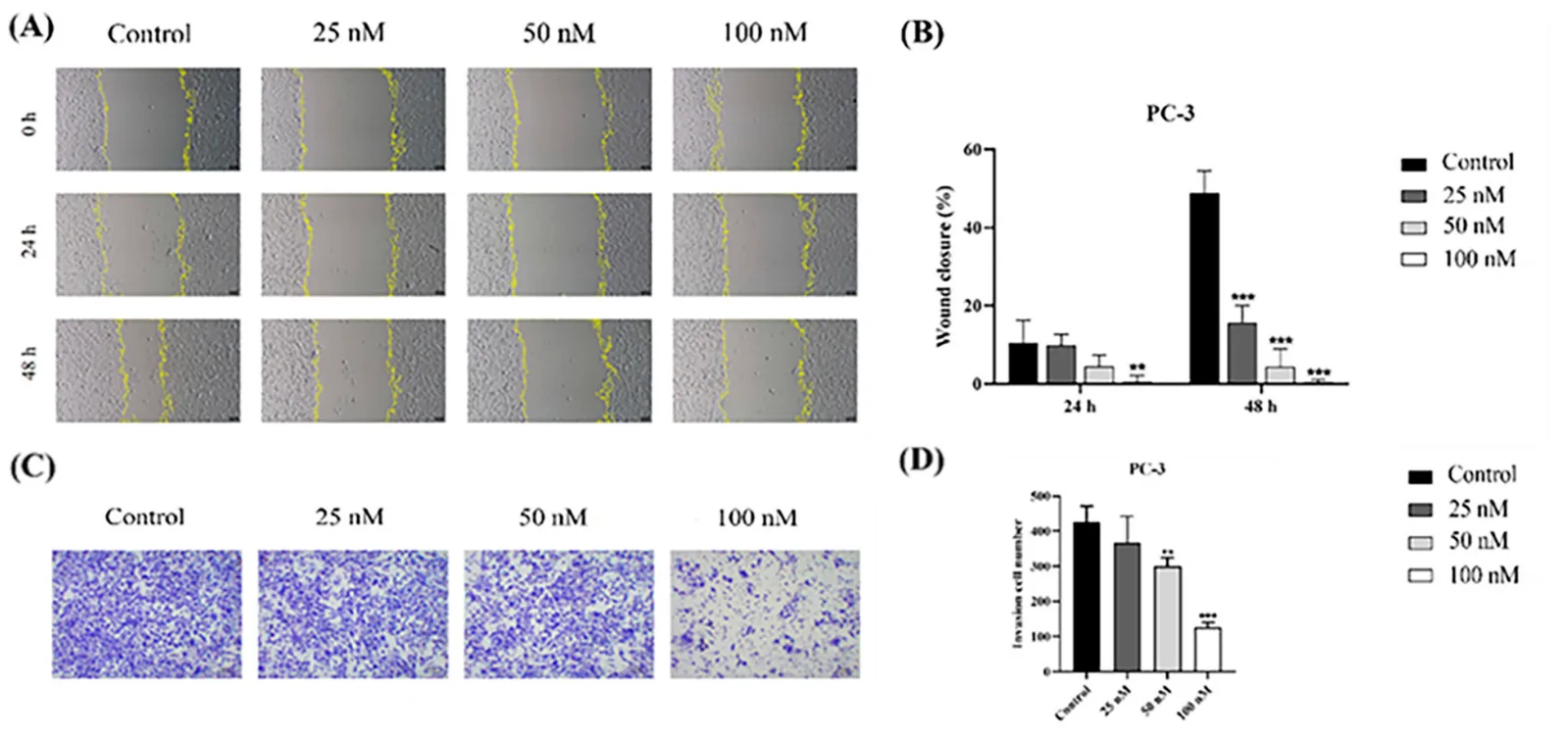
Effects of HHT on the migration and invasion of PCa cells: (**A**) effect of HHT on the migratory ability of PCa cells after 24 and 48 h of treatment; (**B**) statistical histogram of the migration assay; (**C**) effect of HHT on the invasive ability of PCa cells after 48 h of treatment; (**D**) statistical histogram of the invasion assay. Data are presented as mean ± SD. ** *p* < 0.01, *** *p* < 0.001, *n* = 3.

To investigate whether HHT inhibits the migration and invasion of PCa cells, the wound healing assay was employed to evaluate its effect on cell migration. As shown in Figure 4A,B, treatment with 100 nM HHT for 24 h significantly inhibited the migration of PC-3 cells, while after 48 h, all tested concentrations (25, 50, and 100 nM) significantly suppressed PC-3 cell migration. Transwell assays were performed to quantify the effect of HHT on cell invasion. After 48 h of treatment with varying concentrations of HHT, the invasive capacity of cells was markedly decreased in a concentration-dependent manner (Figure 4C,D).

### 3.4. HHT Induces G_1_/S-Phase Arrest in PCa Cells

**Figure 5:**
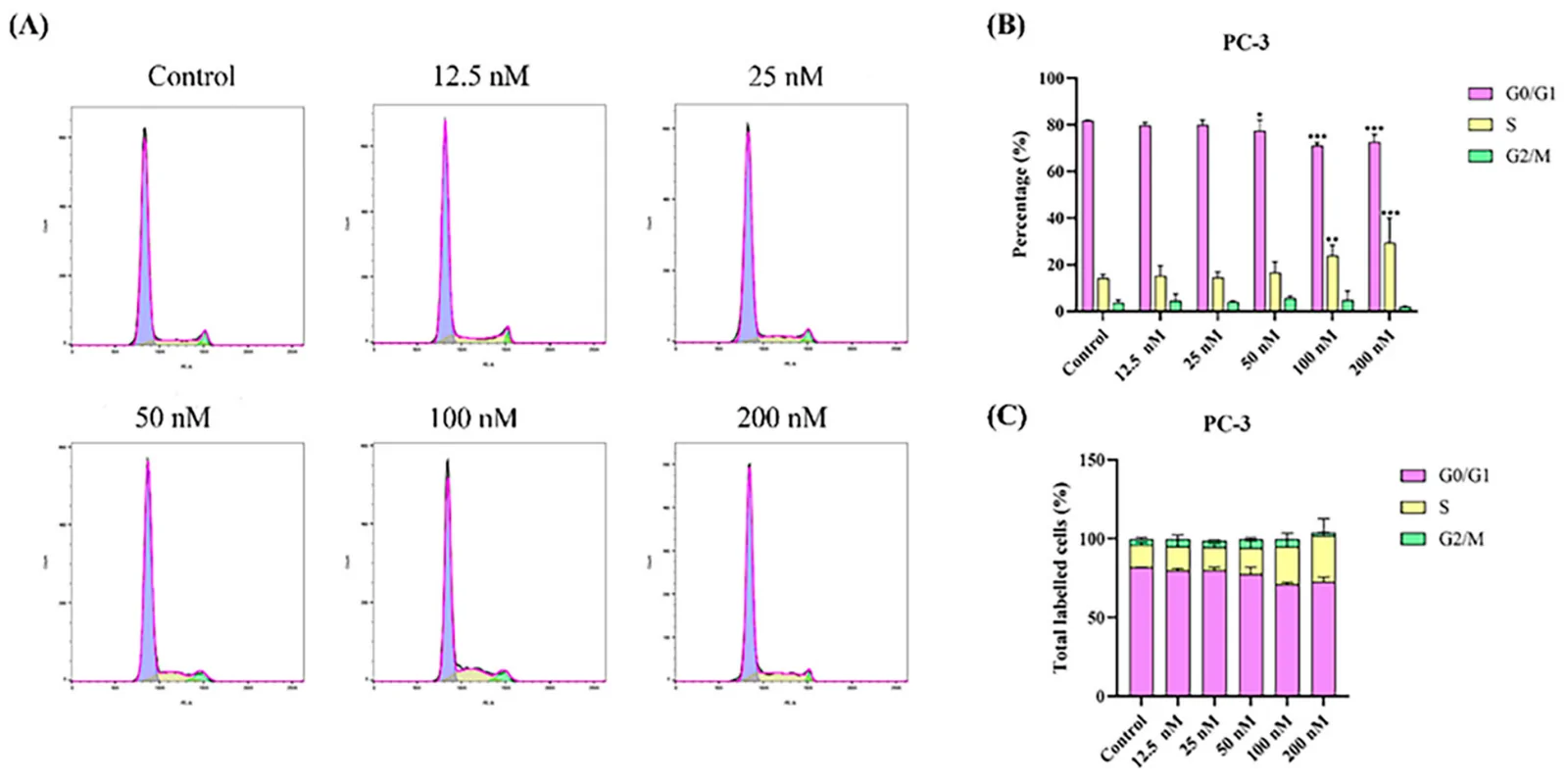
HHT induces cell cycle arrest in PCa cells: (**A**) quantification of the percentage of PC-3 cells in each phase of the cell cycle by flow cytometry after treatment with different concentrations of HHT for 48 h; (**B**) statistical histogram of the cell cycle distribution; (**C**) Stacked composition chart of the overall cell cycle. Data are presented as mean ± SD. * *p* < 0.05, ** *p* < 0.01,*** *p* < 0.001, *n* = 3.

The cell cycle is a highly ordered and precisely regulated process; any abnormality or arrest at any phase can lead to cell cycle restriction and stagnation. Therefore, we investigated whether HHT inhibits cell cycle progression in PCa cells by determining the proportion of cells in each phase of the cell cycle via flow cytometry. With increasing concentrations of HHT, the proportion of PC-3 cells in S phase significantly increased in a dose-dependent manner, particularly at 100 and 200 nM (Figure 5A,B). These results indicate that PC-3 cells failed to complete the normal transition from G1 to S phase, which is consistent with the observed inhibition of cell proliferation described above.

### 3.5. HHT Induces Apoptosis in PCa Cells

**Figure 6:**
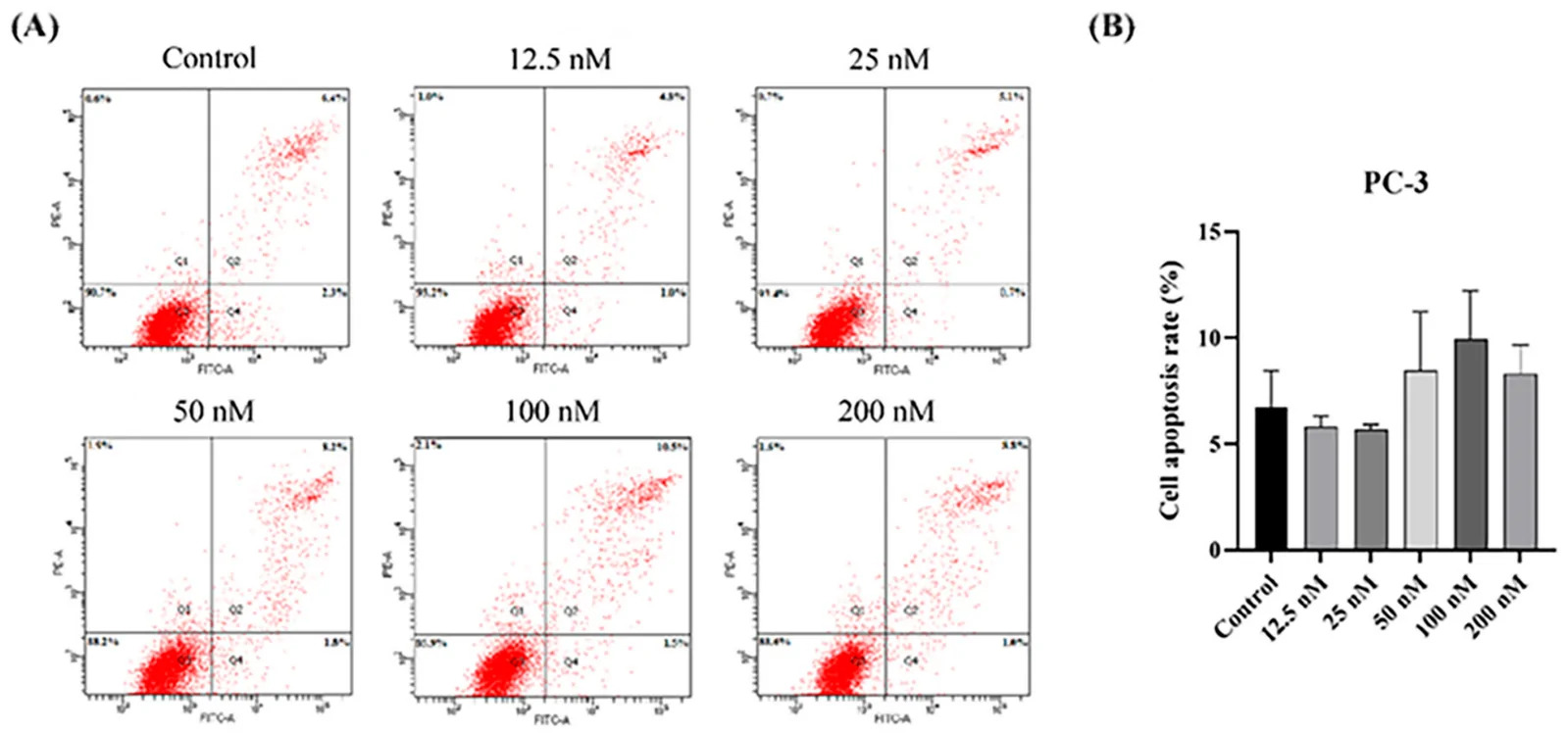
HHT induces apoptosis in PCa cells: (**A**) apoptosis analysis of PC-3 cells by flow cytometry after treatment with different concentrations of HHT for 48 h; (**B**) statistical histogram of apoptosis. Data are presented as mean ± SD. *n* = 3.

To elucidate the effect of HHT on apoptosis of PCa cells, Annexin V-FITC/PI staining combined with flow cytometry was employed to quantitatively analyze apoptosis in PC-3 cells. As shown in Figure 6A,B, HHT treatment increased the proportion of Annexin V-positive cells to a certain extent.

### 3.6. HHT Inhibits PCa Cell Proliferation by Regulating the TNF/NFκB Signaling Pathway

**Figure 7:**
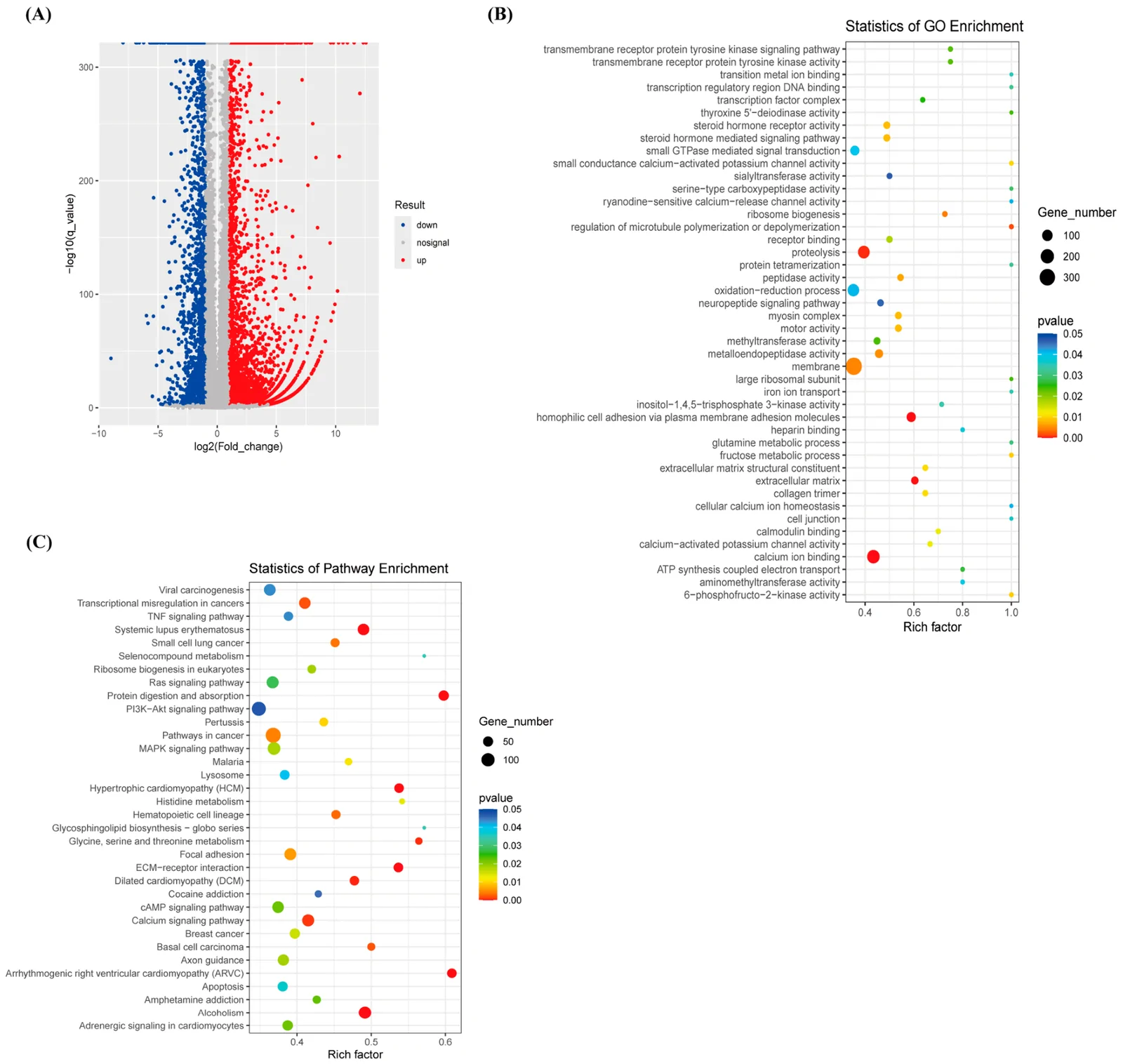
HHT inhibits the TNF/NF-κB and MAPK signaling pathways in PC-3 cells. (**A**) Volcano plot of differentially expressed genes. (**B**) GO enrichment analysis. (**C**) KEGG enrichment analysis.

**Figure 8:**
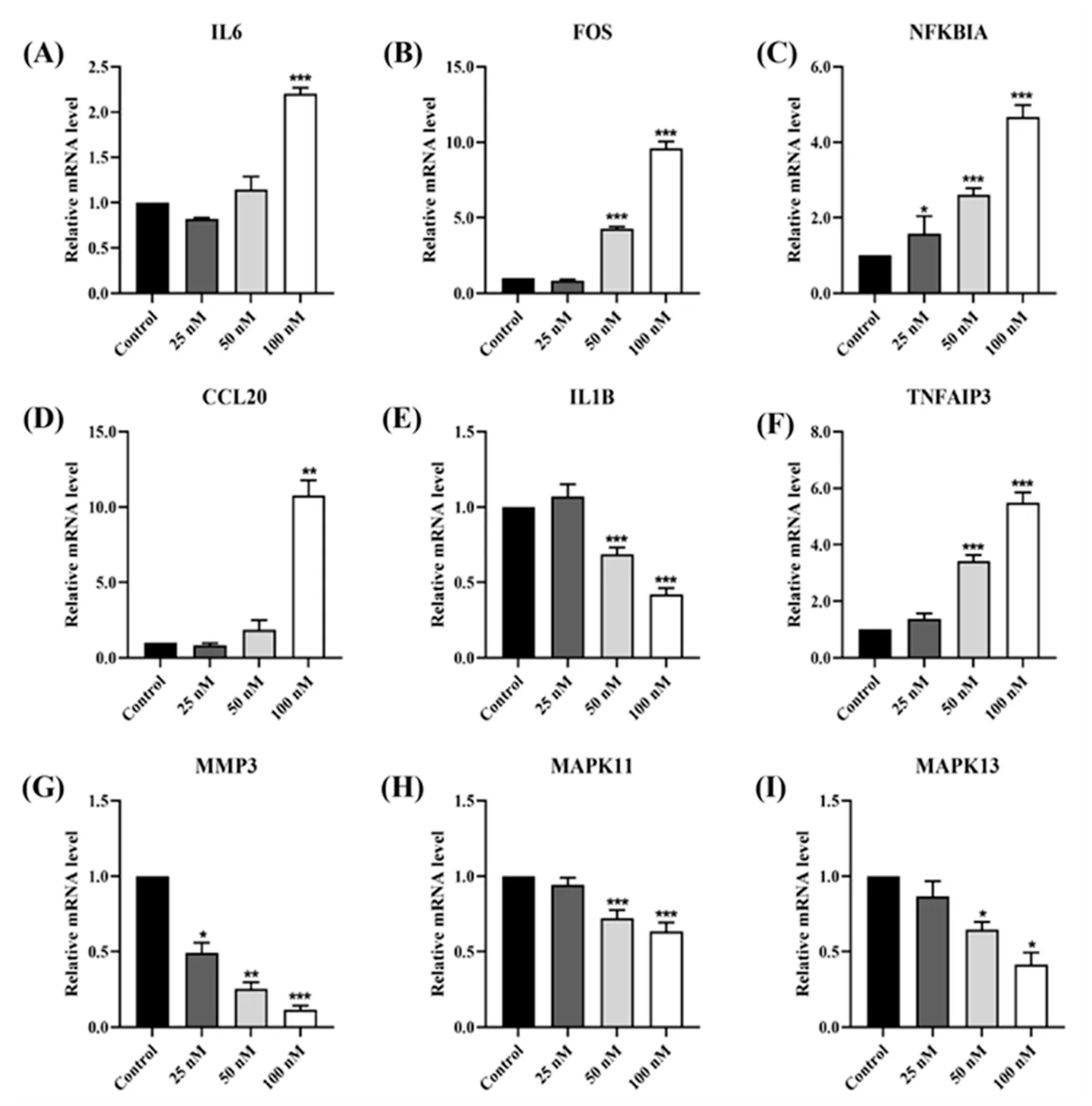
HHT regulates the expression of key genes in the TNF/NF-κB and MAPK signaling pathways. (**A**–**I**) RT-qPCR analysis of gene expression changes. Data are presented as mean ± SD. * *p* < 0.05, ** *p* < 0.01,*** *p* < 0.001, *n* = 3.

To elucidate the mechanism by which HHT inhibits PCa cell proliferation, RNA-seq analysis was performed to examine gene expression profiles. As shown in Figure 7A, a total of 1542 differentially expressed genes (DEGs) were identified, among which 620 were upregulated and 922 were downregulated. Gene Ontology (GO) and Kyoto Encyclopedia of Genes and Genomes (KEGG) enrichment analyses revealed that HHT may exert its anticancer effects by regulating signaling pathways closely associated with tumor progression, such as the TNF and MAPK pathways (Figure 7B,C). To validate the results of the pathway analysis, key genes involved in these pathways were selected, and their expression changes in PC-3 cells were examined by RT-qPCR. As shown in Figure 8, compared with the control group, HHT treatment significantly upregulated the mRNA expression levels of IL-6, FOS, NFKBIA, CCL20, and TNFAIP3, while significantly downregulating the mRNA expression levels of IL1B, MMP3, MAPK11, and MAPK13.

## 4. Discussion

In this study, we found that HHT effectively inhibited PCa cell proliferation and induced apoptosis. This effect may be closely associated with its regulation of the TNF/NF-κB and MAPK signaling pathways. The NF-κB pathway is one of the core inflammatory signaling axes driving PCa progression, and its sustained activation promotes tumor cell survival, proliferation, and chemoresistance [18]. In the canonical pathway, IκBα sequesters the NF-κB dimer in the cytoplasm; upon cellular stimulation, IκBα is phosphorylated and subsequently degraded via the ubiquitin–proteasome pathway, releasing NF-κB transcription factors (e.g., p65/p50) to translocate into the nucleus and initiate transcription [19]. Notably, the NF-κB pathway is subject to tight negative feedback regulation, and one of its target genes, NFKBIA (encoding IκBα), is a classic inhibitory molecule of the NF-κB pathway [19]. Upregulation of NFKBIA usually indicates activation of the upstream pathway, and its significant upregulation implies initiation of a negative feedback loop to limit excessive NF-κB activation [20]. Another key negative regulator, TNFAIP3 (A20, tumor necrosis factor alpha-induced protein 3), terminates TNF receptor- and Toll-like receptor-mediated inflammatory signaling through its deubiquitinating activity [18,21].

HHT effectively limits NF-κB activation by enhancing the negative feedback regulatory mechanism of the NF-κB signaling pathway. Functionally, this is evidenced by the significant downregulation of the NF-κB target genes IL-1β and MMP3. Interleukin-1β (IL-1β) is a potent pro-inflammatory cytokine that plays a dominant regulatory role in immune modulation. Its expression is upregulated in various types of cancer, promoting angiogenesis, metastasis, and immunosuppression [12,22]. MMP3 directly degrades the extracellular matrix and is a key enzyme in tumor metastasis [23]. The decreased expression levels of both genes are consistent with the observed inhibition of cell migration and invasion.

Concurrently, HHT significantly suppressed the MAPK cascade, as evidenced by the reduced expression of MAPK11 (p38β) and MAPK13 (p38δ). The p38 MAPK family (including p38α, p38β, p38γ, and p38δ) plays an important role in promoting cancer progression and chemoresistance [24,25]. Upon activation, p38 MAPK regulates the expression of inflammatory response-, cell cycle-, and apoptosis-related factors by phosphorylating downstream transcription factors such as ATF-2 and C/EBPβ [22,26,27]. Inhibition of p38 activity reduces the phosphorylation level of C/EBPβ, thereby decreasing the transcriptional level of IL-1β [28,29]. Moreover, p38 MAPK-mediated reduction in mRNA stability further diminishes IL-1β expression [30].

Notably, the differential sensitivity of LNCaP and PC-3 cells to HHT may be attributed to their distinct molecular characteristics. LNCaP is AR-positive and hormone-dependent, whereas PC-3 is AR-negative and castration-resistant [31]. As a translation elongation inhibitor, HHT preferentially affects short-lived proteins involved in proliferation and survival, such as c-Myc and Mcl-1 [32]. The dependence of LNCaP on AR signaling and its high demand for protein synthesis may render it more sensitive to HHT. In addition, differences in the status of tumor suppressors such as TP53 and PTEN between the two cell lines may also influence drug response. Our study primarily focuses on castration-resistant prostate cancer; however, these hypotheses regarding other subtypes warrant further experimental validation [33].

## 5. Conclusions

In summary, we found that HHT significantly inhibited tumor growth in vitro without notable adverse effects. HHT impedes PCa progression by regulating the TNF/NF-κB and MAPK signaling pathways, leading to the inhibition of cell proliferation, migration, and invasion; promotion of apoptosis; and induction of G1/S-phase cell cycle arrest. Therefore, our data indicate that HHT exhibits anticancer activity and may serve as a potential candidate drug for PCa treatment. 

## Figures and Tables

**Table 1:** Primer sequences for RT-qPCR.

Genes	Forward Primer(5′-3′)	Reverse Primer(5′-3′)
IL-6	CTTCGGTCCAGTTGCCTTCT	TGGAATCTTCTCCTGGGGGT
IL1B	GTTCTTTGAAGCTGATGGCCC	GAAGCCCTTGCTGTAGTGGT
FOS	CAAGCGGAGACAGACCAACT	GTGAGCTGCCAGGATGAACT
NFKBIA	CCACTCCACTTGGCTGTGAT	TTCCTCGAAAGTCTCGGAGC
CCL20	GCGAATCAGAAGCAAGCAAC	CCGTGTGAAGCCCACAATAAA
TNFAIP3	TCCACAAAGCCCTCATCGAC	TTCGTTTTCAGCGCCACAAG
MAPK11	AGAACGTCATCGGGCTTCT	TGGCACTTGACGATGTTGTT
MAPK13	AAGACCTACGTGTCCCCGA	TCGGCTCAGCTTCTTGATGG
MMP3	ATCCTACTGTTGCTGTGCGT	GGTTCATGCTGGTGTCCTCA
GAPDH	TCCAAAATCAAGTGGGGCGA	AAATGAGCCCCAGCCTTCTC

**Table 2:** IC_50_ values of HHT in human PCa cell lines PC-3 and LNCaP.

Cell Lines	IC_50_ (24 h)	IC_50_ (48 h)	IC_50_ (72 h)
PC-3	67.23	32.31	22.74
LNCaP	23.91	17.77	16.06

## Data Availability

The original contributions presented in this study are included in the article. Further inquiries can be directed to the corresponding author.
